# Mass spectrometry-based, label-free quantitative proteomics of round spermatids in mice

**DOI:** 10.3892/mmr.2014.2460

**Published:** 2014-08-06

**Authors:** HAILONG WANG, YAN LI, LIJUAN YANG, BAOFENG YU, PING YAN, MIN PANG, XIAOBING LI, HONG YANG, GUOPING ZHENG, JUN XIE, RUI GUO

**Affiliations:** 1 Department of Biochemistry and Molecular Biology, Shanxi Medical University, Taiyuan, Shanxi 030001, P.R. China; 2 Fan-Xing Biological Technology Co., Ltd., Beijing 010000, P.R. China; 3 Respiratory Department, The First Affiliated Hospital, Shanxi Medical University, Taiyuan, Shanxi 030001, P.R. China; 4 Centre for Transplantation and Renal Research, The University of Sydney at Westmead Millennium Institute, Sydney, NSW 2145, Australia

**Keywords:** proteome, round spermatid, label-free quantification, spermatogenesis

## Abstract

Round haploid spermatids are formed at the completion of meiosis. These spermatids then undergo morphological and cytological changes during spermiogenesis. Although sperm proteomes have been extensively studied, relatively few studies have specifically investigated the proteome of round spermatids. We developed a label-free quantitative method in combination with 2D-nano-LC-ESI-MS/MS to investigate the proteome of round spermatids in mice. Analysis of the proteomic data identified 2,331 proteins in the round spermatids. Functional classification of the proteins based on Gene Ontology terms and enrichment analysis further revealed the following: 504 of the identified proteins are predicted to be involved in the generation of precursor metabolites and energy; 343 proteins in translation and protein targeting; 298 proteins in nucleotide and nucleic acid metabolism; 275 and 289 proteins in transport and cellular component organization, respectively. A number of the identified proteins were associated with cytoskeleton organization (183), protein degradation (116) and response to stimulus (115). KEGG pathway analysis identified 68 proteins that are annotated as components of the ribosomal pathway and 17 proteins were related to aminoacyl-tRNA biosynthesis. The round spermatids also contained 28 proteins involved in the proteasome pathway and 40 proteins in the lysosome pathway. A total of 60 proteins were annotated as parts of the spliceosome pathway, in which heterogeneous nuclear RNA is converted to mRNA. Approximately 94 proteins were identified as actin-binding proteins, involved in the regulation of the actin cytoskeleton. In conclusion, using a label-free shotgun proteomic approach, we identified numerous proteins associated with spermiogenesis in round spermatids.

## Introduction

Mammalian spermatogenesis is a complex and highly ordered process, in which a diploid progenitor germ cell transforms to highly specialised spermatozoa. This process involves successive mitotic, meiotic and post-meiotic phases. Once meiosis is completed, haploid germ cells termed ‘round spermatids’ are produced; these spermatids subsequently undergo a series of differentiation steps collectively known as ‘spermiogenesis’. In spermiogenesis, round spermatids develop a distinct head, midpiece and tail region; round spermatids also undergo chromatin remodelling, develop an acrosome and become almost completely devoid of cytoplasm. These changes lead to the formation of slender, elongated, mature spermatids, which are released into the lumen of the seminiferous tubule during spermiation (1).

Round haploid spermatids initiate spermiogenesis; successful spermiogenesis is necessary for fertilization, and alterations of this process constitute an important cause of male infertility. This process requires a precise and well coordinated system that regulates the constantly changing patterns of gene and protein expression (2). Therefore, the identification of proteins present in the spermatids can not only provide insights into the molecular basis of spemiogenesis, but also facilitate the identification of cell-specific targets for the diagnosis or induction of male infertility.

Numerous genes involved in spermatogenesis have been identified by differential display (3), serial analysis of gene expression (SAGE) (4) and microarray methods (5). Nevertheless, these methods do not provide pivotal information on the post-transcriptional control of gene expression, changes in protein expression levels and/or protein modifications. In this context, proteomics research has emerged and enhanced our knowledge on cell behavior at the system level, by revealing global patterns of protein content, modification and activity during development (6). Experiments have also been conducted to initiate differential protein expression profiling studies and/or systematic analyses of testicular proteomes in entire organs or isolated spermatogenic cells from various species. Several groups have focused on sperm proteomes, and identified numerous proteins that characterise sperm cells in different mammals (7–11). Although proteomic analyses of the sperm and of different developmental stages of the testis have been performed in different mammalian species, the protein expression profiles of spermiogenesis, particularly of round spermatids, remain unclear.

Mass spectrometry (MS)-based proteomics technology is a powerful tool for large-scale protein identification and quantification (12). Previous proteomic studies have used techniques such as two-dimensional (2-D) polyacrylamide gel electrophoresis (PAGE) and 1-D PAGE of the extracted protein mixture prior to liquid chromatography (LC)-MS/MS identification. Although these techniques require the reduction of sample complexity prior to LC-MS/MS analysis, proteins present in small amounts may not be detectable on the gel, thereby limiting the capacity of MS to identify a number of protein components. A number of quantitative proteomic methods have been developed, including stable isotope labeling and label-free methods (11). The latter is applicable in complex biological systems; in addition, this technique has a number of advantages, such as faster, cleaner and simpler results (13,14). Numerous researchers have employed label-free shotgun proteomic techniques (15–17).

Animal models are commonly used to study the molecular regulation of spermatogenesis. Numerous murine models have been established and applied to study the genes that are up- or downregulated in spermatogenesis. Biologically, meiosis and spermiogenesis are quite similar processes between humans and rodents. In the current study, label-free quantitative shotgun proteomics and mass spectrometry were combined to investigate the protein content of the round spermatids of mice, in order to provide new insights into the molecular regulation of spermiogenesis.

## Materials and methods

### Sample preparation

Round spermatids were isolated according to a previously described method (18) with slight modifications. In the first wave of mouse spermatogenesis, different spermatogenic cells were found at specific time points (days 6, 9, 14, 21, 35 and 60 postpartum). Based on the majority of germ cell types, male mice of different ages are commonly used to isolate differently developed stages of spermatogenic cell types. In this study, ten 35-day old male Balb/c mice were used to isolate round spermatids. The mice were anesthetized with CO_2_ and sacrificed by cervical dislocation. The testes were then removed and decapsulated. The tubulous tissue was cut into small pieces and incubated in 5 ml of phosphate-buffered saline (PBS) containing 0.5 mg/ml of collagenase (Sigma-Aldrich, St. Louis, MO, USA) with continuous agitation at 33°C for 15 min. The dispersed seminiferous cords and cells were allowed to sediment for 5 min and the supernatant was decanted. The pellet was resuspended in 5 ml of PBS containing 0.5 mg/ml of trypsin (Sigma-Aldrich) and 1 μg/ml of DNase (Promega, Madison, WI, USA), and incubated under the same conditions for 15 min. The tissue was dissociated to disperse seminiferous cells by gently pipetting with a Pasteur pipette; the cell suspension was then centrifuged at 80 × g for 10 min. The pellet was washed twice with PBS, filtered using a filter cloth (200 mesh) and resuspended in 20 ml of PBS solution containing 0.5% bovine serum albumin (BSA).

A total of 10^8^ cells were bottom-loaded in a cell separator apparatus with a 12.5 cm diameter (TH-300A; Shanghai Huxi Analysis Instrument Factory Co., Ltd., Shanghai, China) and then incubated in a 2–4% BSA linear gradient in RPMI-1640 medium (Gibco, Grand Island, NY, USA). After 3 h of velocity sedimentation at unit gravity, the cell fractions (10 ml/fraction) were collected from the bottom of the separator apparatus at a rate of 5 ml/min. The cell types, in terms of diameter and morphological characteristics, as well as the purity of each fraction, were assessed under a light microscope (BX43; Olympus Co., Tokyo, Japan). Only fractions with the expected cell type were pooled together. The average purity of round spermatids was 95%.

### Protein extraction

Cells were washed twice with PBS and then lysed by sonication on ice in a buffer containing 7 M urea, 2 M thiourea, 4% CHAPS, 65 mM DTT and 0.2% Biolyte (Bio-Rad, Richmond, CA, USA). Following sonication, the lysates were centrifuged (10,000 × g, 1 h at 4°C), and the supernatants were collected. The protein concentration of each supernatant was assayed using a standard Bradford protein assay kit (Bio-Rad). Approximately 100 μg of the protein sample was reduced using 10 mM DTT at 37°C for 2.5 h, and alkylated with 50 mM iodoacetamide (both from Sigma-Aldrich) at room temperature for 40 min. The sample was then diluted in a solution of 50 mM NH_4_HCO_3_ (Sigma-Aldrich). The protein mixture was digested by incubating in grade-modified trypsin (Promega) at a 1:50 enzyme:protein ratio, at 37°C for 20 h. The tryptic peptide mixture was lyophilized and stored at −80°C until use.

### Immunofluorescent detection

Cells were attached to poly-L-lysine coated microscopy coverslips and were fixed with 2% formaldehyde in microtubule-stabilizing buffer (50 mmol/l PIPES, 5 mmol/l EGTA and 5 mmol/l MgSO_4_) for 1 h. Coverslips were rinsed in PBS and permeabilized for 1 h in 1% Triton X-100 in PBS. Nonspecific antibody binding was prevented by incubation for 1 h in 10% normal goat serum. Microtubules were labeled with anti-α-tubulin monoclonal (Sigma-Aldrich). Primary antibodies were detected using FITC-conjugated rabbit anti-mouse immunoglobulin (Jackson ImmunoResearch Inc., West Grove, PA, USA). DNA was detected by labeling with DAPI. The coverslips were mounted in a drop of VectaShield mounting medium (Vector Laboratories Inc, Burlingame, CA, USA). Coverslips were examined using BX43 Epifluorescence microscope (Olympus Co.).

### Automated 2D-nano-LC-ESI-MS/MS peptide analysis

The extracted peptides were desalted using 1.3 ml C18 solid-phase extraction column (Sep-Pak^®^ Cartridge; Waters Corp., Milford, MA, USA). The peptides were dried using a vacuum centrifuge and were resuspended in loading buffer containing 5 mM ammonium formate (NH_4_FA) and 5% acetonitrile at pH 3.0, Next, peptides were separated and analyzed by 2D strong cation-exchange (SCX)/reversed-phase (RP) nano-scale LC/MS. The experiments were performed on a Nano Aquity UPLC system (Waters Corp.) connected to an LTQ Orbitrap XL mass spectrometer (Thermo Electron Corp., Bremen, Germany) equipped with an online nano-electrospray ion source (Bruker, Auburn, CA, USA).

A 180 μm × 2.4 cm SCX column (Waters Corp), which was packed with 5 μm of polysulfoethyl aspartamide (PolyLC Inc., Columbia, MD, USA) was used for the first dimension. To recover hydrophobic peptides retained on the SCX column after a conventional salt step gradient, an RP step gradient from 5 to 50% acetonitrile (ACN) was applied to the SCX column. A 15-μl plug was performed at each step of the gradient. The SCX column was cleaned once using 1 M NH_4_FA. The plugs were then loaded onto the SCX column with loading buffer, at a flow rate of 15 μl/min for 6 min. A peptide sample (15 μl) was loaded onto the SCX column prior to injection of the gradient plugs. The eluted peptides were then captured using a trap column (Waters Corp.), and salts were diverted to waste. The trap column (2 cm × 180 μm) was packed with a 5 μm Symmetry^®^ C18 column (Waters Corp.). The RP analytical column (15 cm × 100 μm) was packed with a 1.7 μm bridged ethyl hybrid (BEH) C18 particle (Waters Corp.) and then used for protein separation at the second dimension.

The peptides on the RP analytical column were eluted with a three-step linear gradient, balancing with the 95% A buffer 10 min, then starting from 5 to 40% B in 40 min (A, water with 0.1% formic acid; B, ACN with 0.1% formic acid) and increased up to 80% B in 3 min. Afterwards, this solution was reduced to 5% B for 2 min. The column was left to re-equilibrate for 15 min. The column flow rate was maintained at 500 nl/min and the column temperature was maintained at 35°C. Eluted peptides were ionized at 1.9 kV and the ions were analyzed by an LTQ Orbitrap XL Mass spectrometer (Thermo Fisher Scientific Inc., Marietta, OH, USA).

The LTQ Orbitrap XL mass spectrometer was operated in a data-dependent mode to switch automatically between MS and MS/MS acquisition. Survey full-scan MS spectra with two microscans (300–1800 m/z) were acquired in the Obitrap with a mass resolution of 60,000 at 400 m/z. Ten sequential LTQ-MS/MS scans were then conducted. Dynamic exclusion was used with two repeat counts, 10 sec repeat duration and 90 sec exclusion duration. For MS/MS, precursor ions were activated using a 35% normalized collision energy at the default activation q-value of 0.25.

### Peptide sequencing data analysis

The acquired MS/MS spectra were searched against the IPI mouse.v3.68 fasta-formatted protein database using the SEQUEST v.28 (revision 12) software (Thermo Electron Corp.). To reduce identification of false positives, we appended to the database its decoy version containing the reverse sequences. The search parameters were the following: partial trypsin (KR) cleavage with two missed cleavages; the variable modification was oxidation (M); peptide mass tolerance, 50 ppm; and fragment ion tolerance, 1 Da. The open-source Trans Proteomic Pipeline software (revision 4.0; Institute of Systems Biology, Seattle, WA, USA) was then used to identify proteins based on the corresponding peptide sequences and a ≥95% confidence threshold. The peptides results were filtered by Peptide Prophet (19) with a p-value >0.95 and a Protein Prophet (20) probability of 0.95 was used for the protein identification results.

### Bioinformatic analyses

The predicted cellular localization of the proteins identified in the round spermatids was retrieved based on the information available at the Gene Ontology (GO)project website (http://www.geneontology.org/). Functional classification of the proteins was based on biological process and molecular function GO terms. Assignment of the proteins to signaling pathways was based on information available at the Kyoto Encyclopedia of Genes and Genomes (KEGG) (http://www.genome.jp/kegg/pathway.html) and the BioCarta (http://www.biocarta.com/genes/index.asp) databases. Enrichment analysis for these categorizations was performed with tools available at DAVID Bioinformatics Resources (http://david.abcc.ncifcrf.gov/); DAVID is a web-based application that enables visualization, discovery and analysis of molecular interactions and associations with disease for a given list of genes or proteins.

## Results

### Identification of proteins in round spermatids by shotgun proteomics

**Figure 1:**
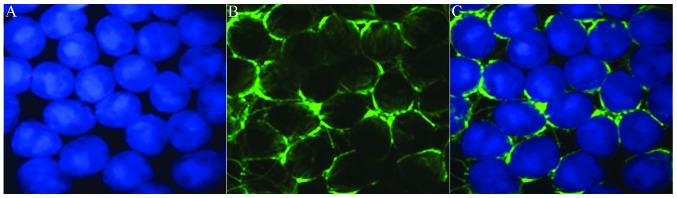
Immunofluorescent staining of purified mouse round spermatids with (A) DAPI (blue), (B) anti-α-tubulin (green), and (C) DAPI and anti-α-tubulin. Strong anti-α-tubulin staining was observed in almost all cells. Scale bar, 20 μm.

Following isolation of murine testicular cells by a gradient method, the purity of the sorted round spermatids was assessed by immunofluorescent staining using the anti-α-tubulin antibody. α-tubulin is the main component of manchette, which is a spermatid-specific microtubular structure. The purity of the sorted round spermatids was >95%, as assessed by counting 500 sorted cells under the microscope (Fig. 1).

We employed a label-free shotgun proteomic technique to identify proteins, gain insights into the protein expression profile of round spermatids, and investigate the relevant molecular mechanisms. The reproducibility of the method was evaluated, with a reliability coefficient of 95% estimated from independent experiments. We found that the peptide spectral intensity is higher than the spectral counts in the quantification of proteomic analysis. The average peptide spectral intensity was used as a standard for the relative quantification of proteins. A total of 2,331 proteins were identified by using the sequenced peptides as queries in searches against the IPI mouse database. Repeating the search against the related decoy database with the same parameters yielded a low (1%) false discovery rate (FDR) at the peptide level, indicating that our approach has high specificity.

### Enriched pathways and functional categories

**Figure 2:**
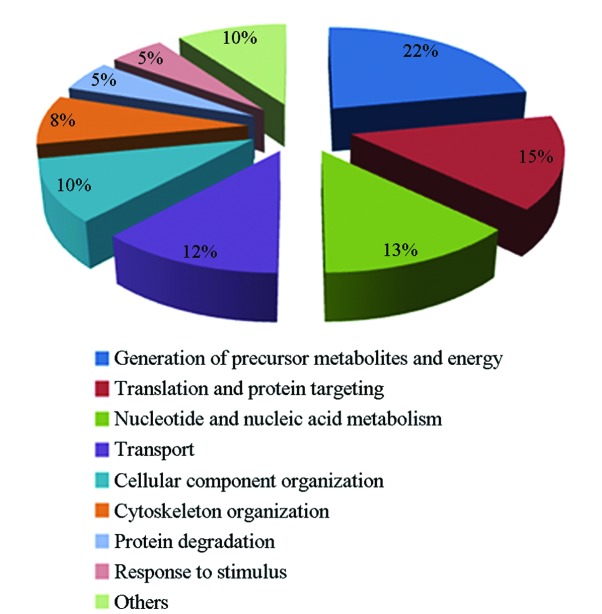
Classification of identified proteins based on relevant biological processes (Gene Ontology terms).

Among the 2,331 identified proteins, 2,287 were found to correspond to unique genes. To characterize these proteins, we initially categorized them based on biological process terms of GO and conducted an enrichment analysis. The most significant categories are shown in Table I. These processes include the generation of precursor metabolites and energy (504), translation and protein targeting (343), nucleotide and nucleic acid metabolism (298), transport (275) and cellular component organization (289). Some of the identified proteins were associated with cytoskeleton organization (183), protein degradation (116) or response to stimulus (115). Approximately 164 proteins with unknown functions were also identified in the proteome of round spermatids. The full classification of the unknown-function proteins with regards to the biological processes they are associated with is demonstrated in a pie chart in Fig. 2.

**Figure 3:**
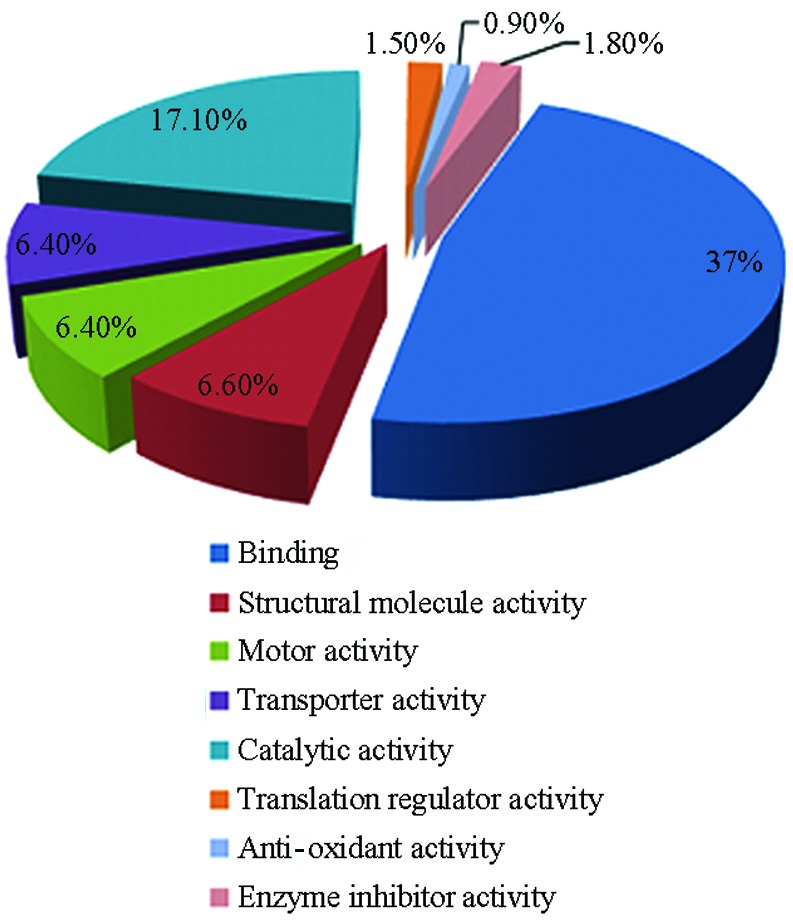
Classification of identified proteins based on relevant molecular functions (Gene Ontology terms).

Furthermore, the predicted molecular function and subcellular localization of the identified proteins was retrieved from GO and enrichment analysis was performed with DAVID tools. A total of 1,818 identified proteins were classified into 9 groups according to their molecular function: binding (866); catalytic activity (400); structural molecule activity (155); motor activity (150); translation regulator activity (35); anti-oxidant activity (19); and enzyme inhibitor activity (43). The full classification of 1,818 proteins is shown in a pie chart in Fig. 3.

**Figure 4:**
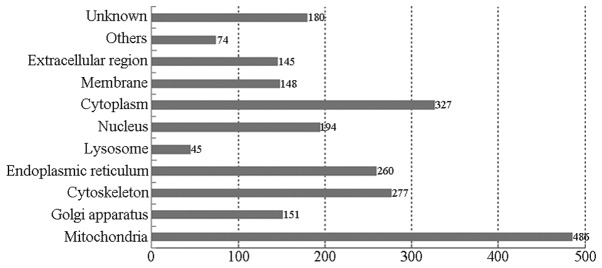
Subcellular localization of identified proteins (DAVID graph).

Fig. 4 shows the classification of the proteins identified in this study according to their predicted subcellular localization. If an individual protein was predicted to localize in more than one cellular compartment, all localizations were counted non-exclusively. The largest proportion of the identified proteins was associated with the mitochondrion (486), followed by the following cell parts/organelles: cytoplasm (327); cytoskeleton (227); endoplasmic reticulum (260); nucleus (194); Golgi apparatus (151); membrane (148); and lysosome (45).

**Figure 5:**
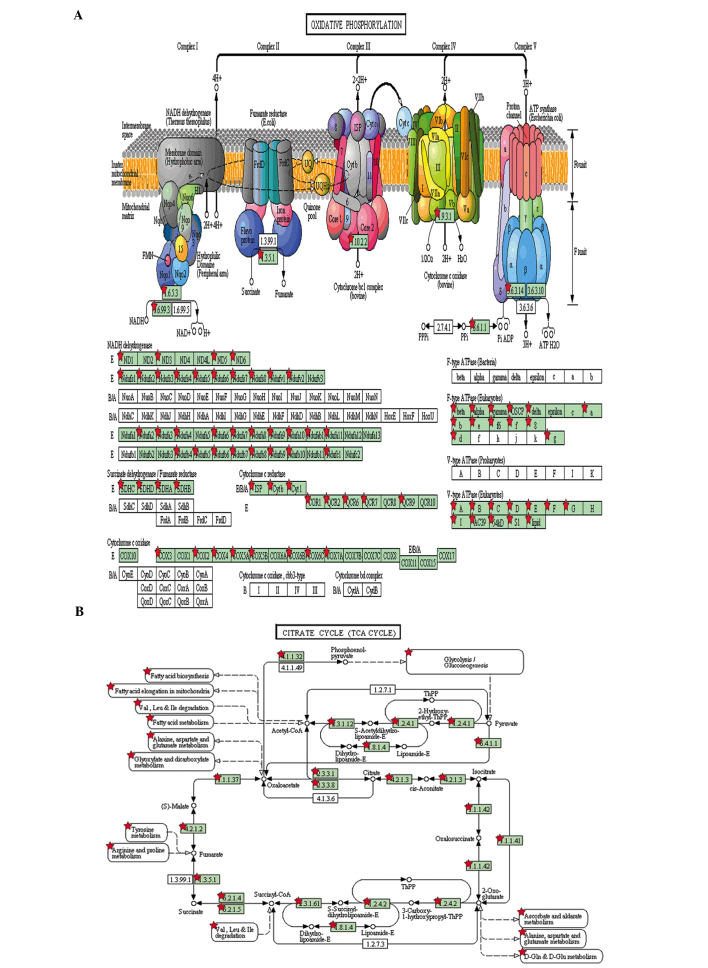
Pathway analysis using the Kyoto Encyclopedia of Genes and Genomes (KEGG) Pathway database. Proteins involved in the (A) oxidative phosphorylation pathway (n=81) and (B) the citrate (TCA) cycle (n=27). Red stars denote the detected proteins.

To investigate the pathways governing the behavior of round spermatids, we further classified the proteins based on KEGG pathway terms. As expected, an important proportion of the identified proteins (370) were involved in metabolic pathways. Among these proteins, 81 were involved in the oxidative phosphorylation pathway (Fig. 5A) that supports spermatid maturation, and 34 were related to the fatty acid metabolism pathway. This pathway provides the necessary energy for spermatid maturation. In addition, 27 proteins were bound to the citric acid (TCA) cycle (Fig. 5B) and 92 proteins were involved in sugar metabolism pathways, such as glycolysis, gluconeogenesis, pyruvate metabolism, starch and sucrose metabolism and the pentose phosphate pathway (data not shown).

**Figure 6:**
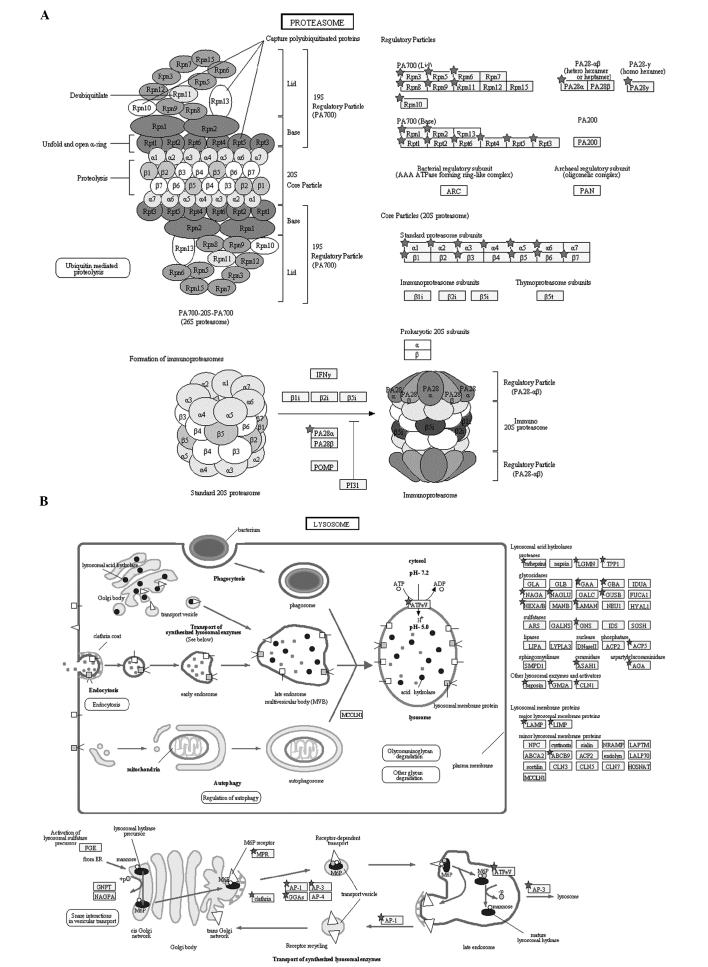
Pathway analysis using the Kyoto Encyclopedia of Genes and Genomes (KEGG) Pathway database. Proteins involved in the (A) proteasome pathway (n=28) and (B) the lysosome pathway (n=40). Stars denote the detected proteins.

**Figure 7:**
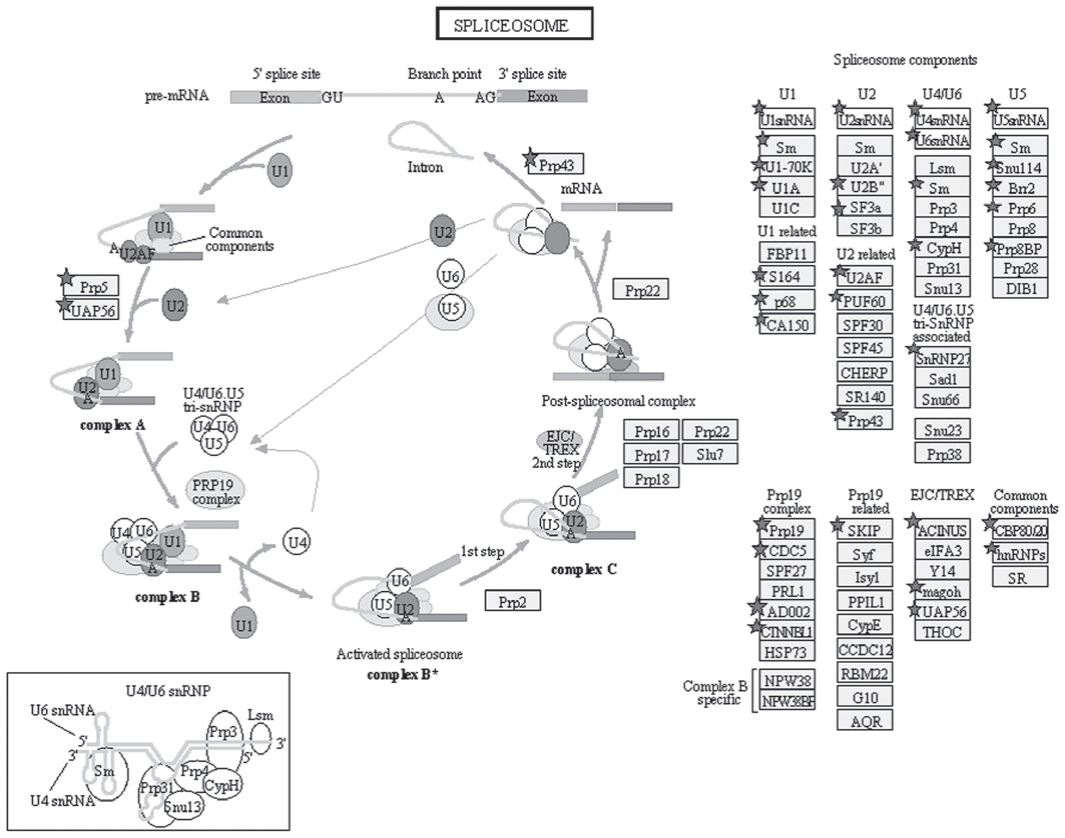
Pathway analysis using the Kyoto Encyclopedia of Genes and Genomes (KEGG) Pathway database. Proteins involved in the spliceosome pathway (n=60) are shown. Stars denote the detected proteins.

In addition to the proteins involved in metabolism, a large group of proteins essential for translation were identified in round spermatids. A total of 68 proteins were annotated as parts of the ribosomal pathway, and 17 proteins as related to aminoacyl-tRNA biosynthesis. Numerous proteins were also involved in protein degradation. We found that the round spermatid proteome contained 28 proteins in the proteasome pathway and 40 proteins in the lysosome pathway (Fig. 6A and B). Pathway annotation of the haploid proteome by the Pathway Studio software (http://www.elsevier.com/online-tools/pathway-studio/pathway-studio-web) revealed that 60 proteins are components of the spliceosome pathway, in which heterogeneous nuclear RNA (hnRNA) is converted to mRNA (Fig. 7).

**Figure 8:**
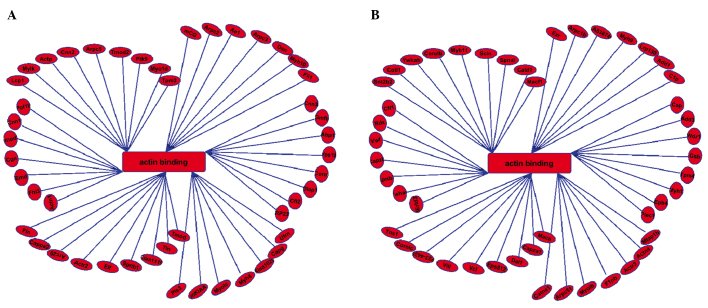
Pathway analysis using the Kyoto Encyclopedia of Genes and Genomes (KEGG) Pathway database. Proteins involved in regulation of the actin cytoskeleton (actin-binding) are shown (n=94).

Numerous actin and actin-binding proteins participate in the formation of sperm. LC-MS/MS analysis performed in this study identified ~94 actin-binding proteins, involved in the regulation of the actin cytoskeleton KEGG pathway in round spermatids of mice (Fig. 8A and B).

**Figure 9:**
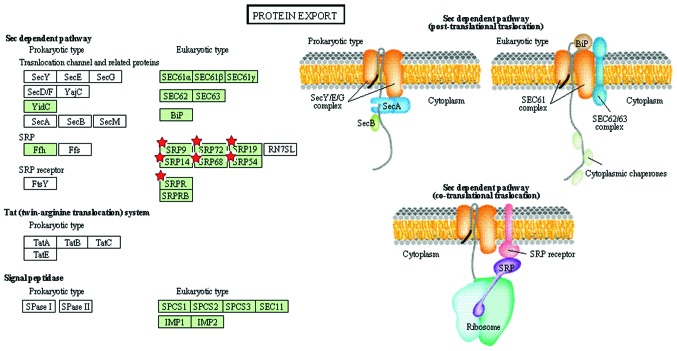
Pathway analysis using the Kyoto Encyclopedia of Genes and Genomes (KEGG) Pathway database. Proteins participating in the nucleocytoplasmic transport pathway (n=7) are shown. Red stars denote the detected proteins.

A total of 25 proteins involved in gap junctions, 44 proteins in tight junctions and 26 proteins in adherens junctions were also detected. Seven proteins involved in the nucleocytoplasmic transport pathway (Fig. 9) and nine proteins related to the caspase cascade of the apoptotic signaling pathway were also identified. Full results from the pathway analysis are shown in Table II.

## Discussion

The proteome of a cell or an organelle provides information regarding the ensemble of proteins expressed in that particular cell or organelle, and the modification of proteins under specific physiological conditions and time points (21). Label-free proteomics is a rapidly growing MS-based quantitative proteomic workflow, since it does not require chemical labeling; quantification is thus unaffected by labelling efficiencies (22). In order to fully characterise spermiogenesis, and in particular the biological characteristics of round spermatids, we obtained, using a label-free proteomic approach, the full proteome of 2,331 proteins of round spermatids of mice; among these proteins, 2,287 mapped to unique genes.

Spermatogenesis is a complex and highly ordered process, which begins with the differentiation of spermatogonial stem cells and ends with the formation of mature sperm. In haploid germ cell differentiation (or spermiogenesis), round spermatids undergo marked morphological changes. The nucleus becomes more compact, the mitochondria are rearranged, the flagellum develops and an acrosome is formed (23). In the present study, β-1-globin, β-2-globin and histone H4 were found to be expressed in round spermatids (data not shown). These proteins are constituents of the chromatin structure and participate in gene regulation (24).

Energy metabolism is a key process for the development of round spermatids. Round spermatids require ATP, most probably to sustain morphological changes, as well as active protein degradation and synthesis. In round spermatids, lactate and pyruvate are the preferred substrates for the generation of energy; the use of glucose is limited (25). In our study, 504 proteins were identified as involved in the generation of precursor metabolites and energy (Table I). The TCA cycle is the main energy resource of round spermatids, although glycolytic and pentose phosphate pathways also contribute to energy production in the spermatids (26). Citrate synthase, isocitrate dehydrogenase and α-oxoglutarate dehydrogenase are expressed in round spermatids (Table I and Fig. 5B). L-lactate dehydrogenase, pyruvate kinase and pyruvate dehydrogenase, which are involved in the glycolytic pathway, are also expressed in round spermatids. Pyruvate kinase is fully activated in round spermatids when glucose is metabolized by the glycolytic pathway (27). A total of 81 proteins were identified as involved in the oxidative phosphorylation pathway in round spermatids (Fig. 5A); these proteins may be involved in the formation and in reactions occurring in the acrosome, which require energy provided by oxidative phosphorylation (25).

At the stage of development of round spermatids, numerous proteins and organelles are degraded; the ubiquitin-proteasome and the lysosome pathways are important, particularly in facilitating the formation of condensed sperms. In the present study, 28 proteins were found as involved in the proteasome pathway and 40 proteins in the lysosome pathway (Fig. 6A and B). Post-translational protein modification by ubiquitination is a signal for lysosomal or proteasomal proteolysis. UBA6 is an E1-activating enzyme, which can activate ubiquitin and FAT10 (28,29). UBA6 uses a specific E2 enzyme, namely, Use1, which cooperates with E3 enzymes to ubiquitylate a unique subset of protein substrates (30). CUL4 is an E3 ubiquitin ligase; in the absence of a functional *CUL4* gene, a decreased number of spermatozoa, reduced sperm motility and defective acrosome formation are observed (31). The ubiquitination of proteins can be regulated and reversed by deubiquitinating enzymes. Ubiquitin C-terminal hydrolases (UCHs) are responsible for the removal of polyubiquitin chains during substrate priming for proteasomal proteolysis. UCHL1 and UCHL3, which were identified in round spermatids in our study, are involved in sperm acrosomal formation and function; these enzymes are known to be important for fertilization (32,33).

Transcription during spermatogenesis begins in almost-round spermatids; these transcripts are then translated during spermatid elongation and acrosome formation (34,35). In our study, 60 proteins were annotated as parts of the spliceosome pathway, in which hnRNA is converted to mRNA and translated to proteins (Fig. 7). Following protein synthesis, some proteins are translocated between the nuclear and cytoplasmic compartments to allow the essential cellular responses to extracellular and intracellular signals. In our study, seven proteins, such as SRP19 and SRP72, were identified as involved in protein transport and regulation of signal transduction (36,37).

Acrosome formation and spermatid nuclear shaping are two major processes of spermiogenesis. Actin and actin-binding proteins are implicated in various aspects, including acrosome formation and nuclear shaping of the spermatids during spermiogenesis. Actin is also involved in germ cell movement, protein transport and nuclear modifications. Numerous actin-binding proteins are found in actin-rich sites, and these proteins bind to actin filaments and modulate their corresponding properties and functions. Myosin, an actin-dependent molecular motor, is involved in a number of important functions in spermiogenesis, such as acrosome biogenesis, vesicle transport, gene transcription and nuclear shaping (38,39). In the current study, ~94 proteins were predicted to be involved in the regulation of the actin cytoskeleton in the round spermatids of mice (Fig. 8).

Numerous studies have focused on the proteomic analysis of spermatogenesis. Nevertheless, current knowledge on the proteome of round spermatids is limited, and the detailed protein patterns of round spermatids remain unknown. Thus, large-scale proteomic approaches such as the one employed in the present study, can provide a rich resource in the study of spermiogenesis, and enrich our knowledge on the biological functions of round spermatids.

In conclusion, this study is the first, to the best of our knowledge, to conduct a proteomic analysis of round spermatids. Round spermatids are formed in a specific phase of spermatogenesis. We performed label-free quantification analysis and identified 2,287 unique proteins, which are involved in energy metabolism, transcription, protein synthesis and degradation, and nucleocytoplasmic transport. These biological processes facilitate the morphological changes to which round spermatids are subjected. The proteome analysis performed in the current study provided a comprehensive characterization of the protein expression profiles of round spermatids. Therefore, the present study is expected to enhance our understanding of the molecular basis of spermatogenesis.

## Figures and Tables

**Table I:** Enriched biological processes in the proteome of round spermatids based on Gene Ontology (GO) terms.

GO id.	Description	Count	%	Q<0.01
GO:0055114	Oxidation reduction	234	10.3356890460	4.40E-58
GO:0008104	Protein localization	198	8.7455830389	1.24E-50
GO:0045184	Establishment of protein localization	180	7.9505300353	4.11E-33
GO:0015031	Protein transport	179	7.9063604240	9.98E-31
GO:0006091	Generation of precursor metabolites and energy	142	6.2720848057	2.54E-24
GO:0046907	Intracellular transport	135	5.9628975265	2.68E-24
GO:0006412	Translation	126	5.5653710247	2.77E-24
GO:0042592	Homeostatic process	114	5.0353356890	5.88E-24
GO:0016192	Vesicle-mediated transport	105	4.6378091873	1.32E-23
GO:0007155	Cell adhesion	100	4.4169611307	1.30E-21
GO:0022610	Biological adhesion	100	4.4169611307	2.13E-21
GO:0034613	Cellular protein localization	97	4.2844522968	7.58E-20
GO:0070727	Cellular macromolecule localization	97	4.2844522968	5.06E-19
GO:0006886	Intracellular protein transport	93	4.1077738516	1.53E-18
GO:0006396	RNA processing	90	3.9752650177	2.20E-18
GO:0044271	Nitrogen compound biosynthetic process	88	3.8869257951	2.54E-18
GO:0016071	mRNA metabolic process	84	3.7102473498	5.99E-18
GO:0055085	Transmembrane transport	83	3.6660777385	1.56E-17
GO:0019725	Cellular homeostasis	81	3.5777385159	5.15E-17
GO:0043933	Macromolecular complex subunit organization	78	3.4452296820	5.78E-17
GO:0006397	mRNA processing	77	3.4010600707	7.10E-16
GO:0065003	Macromolecular complex assembly	76	3.3568904594	1.18E-15
GO:0051186	Cofactor metabolic process	73	3.2243816254	1.27E-15
GO:0007010	Cytoskeleton organization	71	3.1360424028	5.24E-15
GO:0022900	Electron transport chain	67	2.9593639576	8.39E-15
GO:0008380	RNA splicing	67	2.9593639576	1.13E-14
GO:0005996	Monosaccharide metabolic process	64	2.8268551237	1.89E-14
GO:0006732	Coenzyme metabolic process	60	2.6501766784	1.89E-14
GO:0019318	Hexose metabolic process	60	2.6501766784	1.97E-14
GO:0006163	Purine nucleotide metabolic process	59	2.6060070671	1.97E-14
GO:0034654	Nucleobase, nucleoside, nucleotide and nucleic acid biosynthetic process	59	2.6060070671	2.69E-14
GO:0034404	Nucleobase, nucleoside and nucleotide biosynthetic process	59	2.6060070671	3.59E-14
GO:0034621	Cellular macromolecular complex subunit organization	59	2.6060070671	5.07E-14
GO:0009165	Nucleotide biosynthetic process	58	2.5618374558	5.92E-14
GO:0016044	Membrane organization	58	2.5618374558	9.05E-14
GO:0048878	Chemical homeostasis	58	2.5618374558	1.13E-13
GO:0006631	Fatty acid metabolic process	57	2.5176678445	1.46E-13
GO:0034622	Cellular macromolecular complex assembly	57	2.5176678445	1.48E-13
GO:0009259	Ribonucleotide metabolic process	56	2.4734982332	1.53E-13
GO:0009611	Response to wounding	56	2.4734982332	1.89E-13
GO:0009150	Purine ribonucleotide metabolic process	55	2.4293286219	4.22E-13
GO:0032989	Cellular component morphogenesis	55	2.4293286219	4.40E-13
GO:0006006	Glucose metabolic process	54	2.3851590106	5.52E-13
GO:0030030	Cell projection organization	54	2.3851590106	6.21E-13
GO:0015980	Energy derivation by oxidation of organic compounds	53	2.3409893993	7.03E-13
GO:0006164	Purine nucleotide biosynthetic process	53	2.3409893993	1.41E-12
GO:0007264	Small GTPase mediated signal transduction	53	2.3409893993	1.62E-12
GO:0030029	Actin filament-based process	52	2.2968197880	1.65E-12
GO:0009260	Ribonucleotide biosynthetic process	51	2.2526501767	2.84E-12
GO:0006457	Protein folding	51	2.2526501767	4.68E-12
GO:0008610	Lipid biosynthetic process	51	2.2526501767	5.69E-12
GO:0050801	Ion homeostasis	51	2.2526501767	5.93E-12
GO:0009152	Purine ribonucleotide biosynthetic process	50	2.2084805654	6.56E-12
GO:0030036	Actin cytoskeleton organization	50	2.2084805654	7.59E-12
GO:0009141	Nucleoside triphosphate metabolic process	49	2.1643109541	8.27E-12
GO:0009144	Purine nucleoside triphosphate metabolic process	48	2.1201413428	8.27E-12
GO:0009205	Purine ribonucleoside triphosphate metabolic process	47	2.0759717314	9.26E-12
GO:0009199	Ribonucleoside triphosphate metabolic process	47	2.0759717314	1.50E-11
GO:0006461	Protein complex assembly	47	2.0759717314	1.50E-11
GO:0070271	Protein complex biogenesis	47	2.0759717314	1.50E-11
GO:0006873	Cellular ion homeostasis	47	2.0759717314	2.68E-11
GO:0055082	Cellular chemical homeostasis	47	2.0759717314	4.71E-11
GO:0032268	Regulation of cellular protein metabolic process	46	2.0318021201	2.46E-10
GO:0008283	Cell proliferation	45	1.9876325088	2.89E-10
GO:0009145	Purine nucleoside triphosphate biosynthetic process	44	1.9434628975	4.24E-10
GO:0009142	Nucleoside triphosphate biosynthetic process	44	1.9434628975	9.73E-10
GO:0009206	Purine ribonucleoside triphosphate biosynthetic process	43	1.8992932862	1.48E-09
GO:0009201	Ribonucleoside triphosphate biosynthetic process	43	1.8992932862	1.48E-09
GO:0045333	Cellular respiration	42	1.8551236749	1.53E-09
GO:0046034	ATP metabolic process	42	1.8551236749	2.38E-09
GO:0033043	Regulation of organelle organization	42	1.8551236749	2.96E-09
GO:0006605	Protein targeting	41	1.8109540636	5.08E-09
GO:0032535	Regulation of cellular component size	41	1.8109540636	8.13E-09
GO:0006119	Oxidative phosphorylation	39	1.7226148410	9.12E-09
GO:0043062	Extracellular structure organization	39	1.7226148410	1.14E-08
GO:0016052	Carbohydrate catabolic process	38	1.6784452297	1.16E-08
GO:0006754	ATP biosynthetic process	38	1.6784452297	1.21E-08
GO:0006575	Cellular amino acid derivative metabolic process	38	1.6784452297	1.63E-08
GO:0019226	Transmission of nerve impulse	38	1.6784452297	3.36E-08
GO:0007017	Microtubule-based process	37	1.6342756184	3.37E-08
GO:0009719	Response to endogenous stimulus	36	1.5901060071	4.09E-08
GO:0051493	Regulation of cytoskeleton organization	35	1.5459363958	6.21E-08
GO:0046164	Alcohol catabolic process	34	1.5017667845	8.50E-08
GO:0016053	Organic acid biosynthetic process	34	1.5017667845	1.21E-07
GO:0046394	Carboxylic acid biosynthetic process	34	1.5017667845	1.21E-07
GO:0010324	Membrane invagination	34	1.5017667845	1.42E-07
GO:0006897	Endocytosis	34	1.5017667845	1.81E-07
GO:0009725	Response to hormone stimulus	33	1.4575971731	1.85E-07
GO:0007517	Muscle organ development	33	1.4575971731	3.23E-07
GO:0055080	Cation homeostasis	33	1.4575971731	4.24E-07
GO:0044275	Cellular carbohydrate catabolic process	32	1.4134275618	7.66E-07
GO:0008202	Steroid metabolic process	32	1.4134275618	1.18E-06
GO:0007268	Synaptic transmission	32	1.4134275618	1.27E-06
GO:0046395	Carboxylic acid catabolic process	30	1.3250883392	1.31E-06
GO:0016054	Organic acid catabolic process	30	1.3250883392	1.31E-06
GO:0044087	Regulation of cellular component biogenesis	30	1.3250883392	1.49E-06
GO:0006979	Response to oxidative stress	29	1.2809187279	1.49E-06
GO:0033365	Protein localization in organelle	29	1.2809187279	1.50E-06
GO:0030198	Extracellular matrix organization	29	1.2809187279	1.50E-06
GO:0043623	Cellular protein complex assembly	29	1.2809187279	1.50E-06
GO:0055066	Di-, trivalent inorganic cation homeostasis	29	1.2809187279	1.65E-06
GO:0015992	Proton transport	28	1.2367491166	2.14E-06
GO:0019320	Hexose catabolic process	28	1.2367491166	2.24E-06
GO:0006007	Glucose catabolic process	28	1.2367491166	2.36E-06
GO:0006818	Hydrogen transport	28	1.2367491166	2.42E-06
GO:0046365	Monosaccharide catabolic process	28	1.2367491166	3.04E-06
GO:0032956	Regulation of actin cytoskeleton organization	28	1.2367491166	3.40E-06
GO:0032970	Regulation of actin filament-based process	28	1.2367491166	3.88E-06
GO:0045454	Cell redox homeostasis	28	1.2367491166	4.20E-06
GO:0006913	Nucleocytoplasmic transport	28	1.2367491166	4.55E-06
GO:0051169	Nuclear transport	28	1.2367491166	6.02E-06
GO:0051130	Positive regulation of cellular component organization	28	1.2367491166	6.58E-06
GO:0016042	Lipid catabolic process	28	1.2367491166	7.04E-06
GO:0010608	Post-transcriptional regulation of gene expression	28	1.2367491166	8.39E-06
GO:0030003	Cellular cation homeostasis	28	1.2367491166	9.47E-06
GO:0015674	Di-, trivalent inorganic cation transport	28	1.2367491166	1.03E-05
GO:0010035	Response to inorganic substance	27	1.1925795053	1.42E-05
GO:0006790	Sulfur metabolic process	27	1.1925795053	1.43E-05
GO:0006333	Chromatin assembly or disassembly	27	1.1925795053	2.23E-05
GO:0015986	ATP synthesis coupled proton transport	26	1.1484098940	2.39E-05
GO:0015985	Energy coupled proton transport, down electrochemical gradient	26	1.1484098940	2.64E-05
GO:0034220	Ion transmembrane transport	26	1.1484098940	2.89E-05
GO:0008064	Regulation of actin polymerization or depolymerization	26	1.1484098940	4.22E-05
GO:0030832	Regulation of actin filament length	26	1.1484098940	4.27E-05
GO:0043254	Regulation of protein complex assembly	26	1.1484098940	5.34E-05
GO:0051129	Negative regulation of cellular component organization	26	1.1484098940	5.34E-05
GO:0042692	Muscle cell differentiation	26	1.1484098940	5.49E-05
GO:0060537	Muscle tissue development	26	1.1484098940	6.85E-05
GO:0006511	Ubiquitin-dependent protein catabolic process	26	1.1484098940	7.99E-05
GO:0006518	Peptide metabolic process	25	1.1042402827	8.11E-05
GO:0032271	Regulation of protein polymerization	25	1.1042402827	8.43E-05
GO:0014706	Striated muscle tissue development	25	1.1042402827	1.02E-04
GO:0051050	Positive regulation of transport	25	1.1042402827	1.25E-04
GO:0030005	Cellular di-, tri-valent inorganic cation homeostasis	25	1.1042402827	1.79E-04
GO:0006323	DNA packaging	24	1.0600706714	1.90E-04
GO:0042060	Wound healing	24	1.0600706714	1.91E-04
GO:0006084	Acetyl-CoA metabolic process	23	1.0159010601	2.00E-04
GO:0006096	Glycolysis	23	1.0159010601	2.24E-04
GO:0030833	Regulation of actin filament polymerization	23	1.0159010601	2.66E-04
GO:0006399	tRNA metabolic process	23	1.0159010601	2.72E-04
GO:0051187	Cofactor catabolic process	22	0.9717314487	2.96E-04
GO:0043244	Regulation of protein complex disassembly	22	0.9717314487	3.73E-04
GO:0031589	Cell-substrate adhesion	22	0.9717314487	4.24E-04
GO:0051146	Striated muscle cell differentiation	22	0.9717314487	4.24E-04
GO:0051188	Cofactor biosynthetic process	22	0.9717314487	4.87E-04
GO:0007018	Microtubule-based movement	22	0.9717314487	4.87E-04
GO:0022904	Respiratory electron transport chain	21	0.9275618374	4.87E-04
GO:0009109	Coenzyme catabolic process	21	0.9275618374	5.98E-04
GO:0015931	Nucleobase, nucleoside, nucleotide and nucleic acid transport	21	0.9275618374	6.05E-04
GO:0031497	Chromatin assembly	21	0.9275618374	6.14E-04
GO:0065004	Protein-DNA complex assembly	21	0.9275618374	6.22E-04
GO:0017038	Protein import	21	0.9275618374	6.40E-04
GO:0050878	Regulation of body fluid levels	21	0.9275618374	6.65E-04
GO:0005976	Polysaccharide metabolic process	21	0.9275618374	6.65E-04
GO:0009060	Aerobic respiration	20	0.8833922261	6.65E-04
GO:0006418	tRNA aminoacylation for protein translation	20	0.8833922261	8.53E-04
GO:0043039	tRNA aminoacylation	20	0.8833922261	8.84E-04
GO:0043038	Amino acid activation	20	0.8833922261	9.32E-04
GO:0007160	Cell-matrix adhesion	20	0.8833922261	9.32E-04
GO:0007015	Actin filament organization	20	0.8833922261	1.02E-03
GO:0010639	Negative regulation of organelle organization	20	0.8833922261	1.13E-03
GO:0050657	Nucleic acid transport	20	0.8833922261	1.13E-03
GO:0051236	Establishment of RNA localization	20	0.8833922261	1.22E-03
GO:0050658	RNA transport	20	0.8833922261	1.23E-03
GO:0006403	RNA localization	20	0.8833922261	1.32E-03
GO:0006334	Nucleosome assembly	20	0.8833922261	1.42E-03
GO:0034728	Nucleosome organization	20	0.8833922261	1.45E-03
GO:0006099	Tricarboxylic acid cycle	19	0.8392226148	1.46E-03
GO:0046356	Acetyl-CoA catabolic process	19	0.8392226148	1.47E-03
GO:0009064	Glutamine family amino acid metabolic process	19	0.8392226148	1.47E-03
GO:0051494	Negative regulation of cytoskeleton organization	19	0.8392226148	1.71E-03
GO:0019748	Secondary metabolic process	19	0.8392226148	1.84E-03
GO:0030031	Cell projection assembly	19	0.8392226148	2.06E-03
GO:0007599	Hemostasis	19	0.8392226148	2.06E-03
GO:0016125	Sterol metabolic process	19	0.8392226148	2.06E-03
GO:0006417	Regulation of translation	19	0.8392226148	2.06E-03
GO:0043242	Negative regulation of protein complex disassembly	18	0.7950530035	2.06E-03
GO:0006800	Oxygen and reactive oxygen species metabolic process	18	0.7950530035	2.16E-03
GO:0048193	Golgi vesicle transport	18	0.7950530035	2.26E-03
GO:0051028	mRNA transport	18	0.7950530035	2.33E-03
GO:0008203	Cholesterol metabolic process	18	0.7950530035	2.34E-03
GO:0007596	Blood coagulation	18	0.7950530035	2.44E-03
GO:0050817	Coagulation	18	0.7950530035	2.73E-03
GO:0002526	Acute inflammatory response	18	0.7950530035	2.78E-03
GO:0042493	Response to drug	18	0.7950530035	2.84E-03
GO:0006749	Glutathione metabolic process	17	0.7508833922	2.86E-03
GO:0030834	Regulation of actin filament depolymerization	17	0.7508833922	2.86E-03
GO:0044242	Cellular lipid catabolic process	17	0.7508833922	3.04E-03
GO:0051170	Nuclear import	17	0.7508833922	3.19E-03
GO:0055001	Muscle cell development	17	0.7508833922	3.26E-03
GO:0009310	Amine catabolic process	17	0.7508833922	3.28E-03
GO:0009309	Amine biosynthetic process	17	0.7508833922	3.34E-03
GO:0051248	Negative regulation of protein metabolic process	17	0.7508833922	3.34E-03
GO:0006633	Fatty acid biosynthetic process	17	0.7508833922	3.34E-03
GO:0060627	Regulation of vesicle-mediated transport	17	0.7508833922	3.77E-03
GO:0009791	Post-embryonic development	17	0.7508833922	3.99E-03
GO:0018130	Heterocycle biosynthetic process	16	0.7067137809	4.28E-03
GO:0055002	Striated muscle cell development	16	0.7067137809	4.35E-03
GO:0034504	Protein localization in nucleus	16	0.7067137809	4.53E-03
GO:0032269	Negative regulation of cellular protein metabolic process	16	0.7067137809	5.12E-03
GO:0002449	Lymphocyte mediated immunity	16	0.7067137809	5.13E-03
GO:0030835	Negative regulation of actin filament depolymerization	15	0.6625441696	5.13E-03
GO:0032272	Negative regulation of protein polymerization	15	0.6625441696	5.25E-03
GO:0031333	Negative regulation of protein complex assembly	15	0.6625441696	5.46E-03
GO:0000302	Response to reactive oxygen species	15	0.6625441696	5.56E-03
GO:0051258	Protein polymerization	15	0.6625441696	5.56E-03
GO:0009063	Cellular amino acid catabolic process	15	0.6625441696	5.56E-03
GO:0006606	Protein import into nucleus	15	0.6625441696	6.69E-03
GO:0070482	Response to oxygen levels	15	0.6625441696	6.69E-03
GO:0006694	Steroid biosynthetic process	15	0.6625441696	6.69E-03
GO:0042773	ATP synthesis coupled electron transport	14	0.6183745583	6.74E-03
GO:0051693	Actin filament capping	14	0.6183745583	7.11E-03
GO:0042743	Hydrogen peroxide metabolic process	14	0.6183745583	7.27E-039
GO:0034599	Cellular response to oxidative stress	14	0.6183745583	7.31E-03
GO:0042542	Response to hydrogen peroxide	14	0.6183745583	8.12E-03
GO:0030837	Negative regulation of actin filament polymerization	14	0.6183745583	8.13E-03
GO:0034330	Cell junction organization	14	0.6183745583	8.55E-03
GO:0006413	Translational initiation	14	0.6183745583	8.62E-03
GO:0008652	Cellular amino acid biosynthetic process	14	0.6183745583	8.74E-03
GO:0006997	Nucleus organization	14	0.6183745583	9.32E-03

Q, value calculated from p-value by Benjamini-Hochberg-Yekutieli multiple testing correction with Fisher discriminant analysis.

**Table II:** Pathway analysis in the round spermatid proteome using the Kyoto Encyclopedia of Genes and Genomes (KEGG) and the BioCarta Pathway databases.

Source	Term	Count	%	P	Q^a^
KEGG	Ribosome	68	3.0	6.5E-32	1.2E-29
KEGG	Oxidative phosphorylation	81	3.6	3.2E-28	2.9E-26
KEGG	Parkinson’s disease	73	3.2	6.9E-21	4.2E-19
KEGG	Alzheimer’s disease	85	3.8	2.1E-18	9.6E-17
KEGG	Valine, leucine and isoleucine degradation	36	1.6	1.9E-17	7.0E-16
KEGG	Huntington’s disease	83	3.7	4.8E-17	1.5E-15
KEGG	Fatty acid metabolism	34	1.5	1.1E-15	2.9E-14
KEGG	Citrate (TCA) cycle	27	1.2	4.2E-15	9.8E-14
KEGG	Spliceosome	60	2.7	6.8E-14	1.4E-12
KEGG	Glycolysis/Gluconeogenesis	38	1.7	2.3E-11	4.3E-10
KEGG	Propanoate metabolism	23	1.0	7.5E-11	1.3E-9
KEGG	Glutathione metabolism	30	1.3	1.6E-9	2.5E-8
KEGG	Proteasome	28	1.2	2.4E-9	3.5E-8
KEGG	Pyruvate metabolism	25	1.1	1.1E-8	1.5E-7
KEGG	Focal adhesion	66	2.9	5.7E-7	7.0E-6
KEGG	Butanoate metabolism	21	0.9	1.1E-6	1.3E-5
KEGG	ECM-receptor interaction	35	1.5	1.3E-6	1.4E-5
KEGG	Drug metabolism	31	1.4	9.6E-6	9.9E-5
KEGG	PPAR signaling pathway	32	1.4	1.1E-5	1.1E-4
KEGG	Arginine/proline metabolism	24	1.1	2.2E-5	2.1E-4
KEGG	Tight junction	44	1.9	1.0E-4	9.2E-4
KEGG	Lysosome	40	1.8	1.1E-4	9.2E-4
KEGG	Metabolism of xenobiotics by cytochrome P450	26	1.1	1.5E-4	1.2E-3
KEGG	β-alanine metabolism	13	0.6	1.5E-4	1.2E-3
KEGG	Tryptophan metabolism	18	0.8	3.5E-4	2.6E-3
KEGG	Alanine, aspartate and glutamate metabolism	15	0.7	3.7E-4	2.6E-3
KEGG	Protein export	7	0.3	7.7E-4	5.3E-3
KEGG	Fatty acid elongation in mitochondria	7	0.3	7.7E-4	5.3E-3
KEGG	Starch and sucrose metabolism	16	0.7	1.0E-3	6.6E-3
KEGG	Pentose phosphate pathway	13	0.6	1.1E-3	6.9E-3
KEGG	Leukocyte transendothelial migration	37	1.6	1.1E-3	6.8E-3
KEGG	Cardiac muscle contraction	27	1.2	1.1E-3	6.7E-3
KEGG	Valine, leucine and isoleucine biosynthesis	8	0.4	1.2E-3	6.8E-3
KEGG	Porphyrin and chlorophyll metabolism	14	0.6	1.4E-3	7.9E-3
KEGG	Adherens junction	26	1.1	1.7E-3	9.3E-3
KEGG	Aminoacyl-tRNA biosynthesis	17	0.8	2.1E-3	1.1E-2
KEGG	Galactose metabolism	12	0.5	5.8E-3	2.9E-2
KEGG	Regulation of actin cytoskeleton	56	2.5	6.2E-3	3.0E-2
KEGG	Pentose and glucuronate interconversions	9	0.4	6.5E-3	3.1E-2
KEGG	Arrhythmogenic right ventricular cardiomyopathy	24	1.1	6.9E-3	3.2E-2
KEGG	Amino sugar and nucleotide sugar metabolism	16	0.7	9.6E-3	4.4E-2
KEGG	Limonene and pinene degradation	8	0.4	1.2E-2	5.1E-2
KEGG	Ascorbate and aldarate metabolism	8	0.4	1.2E-2	5.1E-2
KEGG	Phenylalanine metabolism	10	0.4	1.2E-2	5.1E-2
KEGG	Long-term potentiation	22	1.0	1.2E-2	5.2E-2
KEGG	Fc γ R-mediated phagocytosis	28	1.2	1.7E-2	6.8E-2
KEGG	Glyoxylate and dicarboxylate metabolism	8	0.4	1.7E-2	6.9E-2
KEGG	Tyrosine metabolism	14	0.6	1.9E-2	7.3E-2
KEGG	Gap junction	25	1.1	2.0E-2	7.5E-2
KEGG	Synthesis and degradation of ketone bodies	6	0.3	2.3E-2	8.7E-2
KEGG	Lysine degradation	14	0.6	2.8E-2	1.0E-1
KEGG	N-glycan biosynthesis	15	0.7	3.3E-2	1.2E-1
KEGG	Prion diseases	12	0.5	4.5E-2	1.5E-1
KEGG	Long-term depression	20	0.9	6.0E-2	2.0E-1
KEGG	Fructose and mannose metabolism	12	0.5	6.5E-2	2.1E-1
KEGG	Oocyte meiosis	29	1.3	6.7E-2	2.1E-1
KEGG	Renin-angiotensin system	7	0.3	9.7E-2	2.9E-1
BioCarta	Shuttle for transfer of acetyl groups from mitochondria to the cytosol	8	0.4	7.9E-5	1.7E-2
BioCarta	uCalpain and friends in cell spread	8	0.4	3.7E-3	3.3E-1
BioCarta	ERAD pathway	9	0.4	6.6E-3	3.8E-1
BioCarta	AKAP95 role in mitosis and chromosome dynamics	6	0.3	1.9E-2	6.4E-1
BioCarta	Agrin in postsynaptic differentiation	11	0.5	2.2E-2	6.1E-1
BioCarta	Cycling of Ran in nucleocytoplasmic transport	4	0.2	2.7E-2	6.2E-1
BioCarta	Protein kinase A at the centrosome	7	0.3	2.9E-2	6.0E-1
BioCarta	Caspase cascade in apoptosis	9	0.4	4.1E-2	6.8E-1
BioCarta	Endocytotic role of NDK, phosphins and dynamin	5	0.2	5.5E-2	7.4E-1
BioCarta	Role of β-arrestins in the activation and targeting of MAP kinases	7	0.3	6.0E-2	7.3E-1
BioCarta	How progesterone initiates the oocyte maturation	8	0.4	8.5E-2	8.2E-1
BioCarta	Role of Ran in mitotic spindle regulation	5	0.2	8.5E-2	7.9E-1
BioCarta	ChREBP regulation by carbohydrates and cAMP	5	0.2	8.5E-2	7.9E-1
BioCarta	CFTR and b2AR pathway	5	0.2	8.5E-2	7.9E-1
BioCarta	Rho-selective guanine exchange factor AKAP13 mediates stress fiber formation	4	0.2	9.8E-2	8.2E-1

a Q, value calculated from p-value by Benjamini-Hochberg-Yekutieli multiple testing correction.

ECM, extracellular matrix; PPAR, peroxisome proliferator-activated receptors; ERAD, endoplasmic-reticulum-associated degradation; AKAP, A-kinase anchoring protein; NDK, nucleoside diphosphate kinase; ChREBP, carbohydrate-responsive element-binding protein; CFTR, cystic fibrosis transmembrane conductance regulator; b2AR, β 2 adrenergic receptor
